# Frontiers in Chronobiology: Endogenous Clocks at the Core of Signaling Pathways in Physiology

**DOI:** 10.3389/fphys.2021.684745

**Published:** 2021-05-20

**Authors:** Rodolfo Costa

**Affiliations:** Department of Biology, University of Padova, Italian National Research Council (CNR) Institute of Neuroscience, Padova, Italy

**Keywords:** endogenous rhythmicity, canonical and non-canonical circadian clocks, photic entrainment, non-circadian clocks, COVID-19

Chronobiology is a relatively young and fast evolving research field, which aims at understanding the origin, the mechanisms and the prerogatives of endogenous biological clocks.

The Chronobiology section of Frontiers in Physiology provides an interdisciplinary forum for the publication of research covering all aspects of the field, including molecular clock circuitry, clock evolution, animal models, physiology, translational studies, and chronotherapy. Over the past few decades, chronobiology has moved from occupying a specialist niche within physiology research, to influencing every aspect at all levels of the discipline. In 2017, the Nobel Prize in Physiology or Medicine was awarded to Jeffrey C. Hall, Michael Rosbash and Michael W. Young, three chronobiologists and drosophilists, “for their discoveries of molecular mechanisms that control circadian rhythms.” The field went on to receive considerably more interest and attention, and the efforts of those researchers who had been working on chronophysiology and its medical implications and applications were also rewarded as chronobiology entered its true translational era (Cederroth et al., 2019). This has been characterized by a flourishing of relevant, novel clinical observations (to name one, the fact that the outcomes of certain types of cardiac surgery are heavily dependent on time of day; Montaigne et al., 2018), by the evolution and the definition of an almost entirely novel chronobiology vocabulary and, most interestingly, by experiments and observations that constantly challenge the few true dogmas of this relatively young science. The definition of clock cells themselves has changed, moving away from the idea that there are cells with specific features that qualify them as oscillators to a model where the clock or oscillator results from the interaction of distinct physiological players (circadian networks) (Mizrak et al., 2012). Similarly, it has become evident that brain structures other than the suprachiasmatic nuclei (SCN) clock neurons [for example astrocytes within the SCN itself (Hastings et al., 2019), the habenula (Baño-Otálora and Piggins, 2017) and the blood brain barrier (Cuddapah et al., 2019)] exhibit clock properties or produce oscillations that modulate SCN outputs in many different ways. Thus rhythmicity, both circadian and over other time scales (for example seasonal, lunar and tidal) is transforming into an ever more complex, versatile and interesting natural phenomenon. The influence of chronobiology on society at large has also been profound, resulting in campaigns, for example, to modify urban lighting, to amend school times and to abolish daylight saving time (Roenneberg et al., 2019). Amongst these developments, I will now focus on a few that fascinate me and, hopefully, will stimulate you.

## In Search of Primordial and Non-Canonical Clocks

Over the past 15 years or so, evidence has accumulated that non-canonical circadian clocks–i.e., clocks which are not based on the transcription/translation feedback loop (TTFL) that characterizes the molecular timing mechanisms of almost all organisms investigated so far–also exist. Further, they seem to play a significant role in orchestrating the temporal expression of portions of the genome in several organisms. For example, the cyanobacterium *Synecochoccus elongatus* exhibits a circadian biochemical oscillation involving three clock proteins (KAI A, KAI B, and KAI C), generating a post-transcriptional phosphorylation loop, which occurs *in vivo* and can be reproduced *in vitro*, in the absence of transcription and translation (Nakajima et al., 2005). More recently, TTFL-independent oxidation-reduction circadian cycles of peroxiredoxins (i.e., highly conserved antioxidant proteins involved in the control of peroxide levels) have been described in bacteria, archaea, fungi, plants and animals (O'Neill and Reddy, 2011; O'Neill et al., 2011; Edgar et al., 2012). Both KAI B and peroxiredoxins belong to the superfamily of thioredoxins and they may represent conserved relics of the primordial clock of the last common ancestor of prokaryotes and eukariotes. While progress has been made in understanding the molecular mechanisms driving these oscillations, their origin and most of their features remain obscure. There are also indications that a non-canonical clock controls the expression of a significant set of genes, proteins and protein modifications in mammalian cells and tissues cultivated *ex vivo* (Ray et al., 2020). In further detail, cultured (i.e., not under the influence of the SCN) skin fibroblasts and liver slices defective for BMAL1, a transcription factor which is essential for the TTFL-based circadian clock, both exhibit 24-h oscillations of portions of their transcriptome, proteome, and phosphoproteome. The authors propose that this insofar ignored piece of clockwork could result from the interplay of a novel set of transcription factors and non-transcriptionally regulated peroxiredoxin-like redox oscillations (Ray et al., 2020). Nonetheless, the findings remain unexpected and concerns have been recently raised in relation to their consistency, validity and significance (Abruzzi et al., 2021; Ness-Cohn et al., 2021). Finally, the examination and interpretation of available data on the role of circadian and non-canonical clocks in embryonic development suggests that cell division, metabolism and epigenetic modifications become temporally organized before the emergence of a functional TTFL clock (Bedont et al., 2020). Thus a non-canonical, somewhat primordial clock would regulate development throughout cell stem progression toward pluripotency. The nature of such clock, the exact temporal definition of a TTFL clock and their respective roles in early cell commitment are one of the hottest topics in the field.

## The True Colors of Circadian Photopigments

The nature of the photopigments and photoreceptors mediating mammalian SCN synchronization with the environment by means of light had been a puzzling issue until Provencio et al. (1998) discovered melanopsin in retinal tissues and hypotesized a role for it in circadian physiology. Then Hattar et al. (2002) and Provencio et al. (2002) went on to describe intrinsically photosensitive retinal ganglion cells (ipRGC), within the inner retina, containing melanopsin and sending monosynaptic projections to the SCN. For a long time the paradigm was that only these cells (about 1% of the all RGCs)–and not the classical photoreceptors rods and cones (contributing to perceptual vision and located in the outer portion of the retina)–contributed to photic entrainment of the master clock through their blue light-sensitive photopigment melanopsin. ipRGCs are less sensitive to light than rods and cones, they are depolarized rather than hyperpolarized by light, and more recently it has also been shown that, in addition to the SCN, they innervate several other areas of the brain, to regulate non-image forming responses to light (Fernandez et al., 2016). These include modulation of melatonin synthesis in the pineal gland, synaptic plasticity in the hippocampus (Fernandez et al., 2016) and functioning of the lateral habenula, which has been implicated in phenotypes such as sleep, mood and propensity to addiction (Baño-Otálora and Piggins, 2017).

More recent studies have pointed to an even more complex ipRGCs form of signaling to the brain, and to the SCN in particular, involving also rods and cones through largely unknown mechanisms but within a neural network which includes bipolar and amacrine cells (Ko, 2020). This model is supported by the observations that melanopsin null mutants mice can still be somehow synchronized by light (Panda et al., 2003) and exhibit phase shifting responses, albeit strongly attenuated (Ruby et al., 2002). Further, photic entrainment is abolished if ipRGCs are completely ablated (Chen et al., 2011). Thus it is ipRGCs and not melanopsin that are essential for photic entrainment, implying that they functionally interact with rods and cones. Therefore, alternative photopigments sensitive to other wavelengths could, through ipRGCs projections, modulate SCN photic entrainment and, most likely, also non-visual light responses depending on other areas of the brain. Finally, inner retina melanopsin, in spite of its low temporal resolution, has also been implicated in some features of form and spatial vision, raising fascinating questions on its role in visual perception (Allen et al., 2019). Thus, time seems ripe for un upgrade of the palette of light colors which modulate non visual photoreception. This will no doubt inform and possibly re-define some aspects of light hygiene over the 24 hours.

## Non-Circadian Rhythmicity

Convincing evidence of the existence of *bona fide* endogenous clocks dictating time in temporal domains other than the circadian one, and information on their molecular and functional features are recent acquisitions. Evidence for circatidal, circalunar, circannual and seasonal biological rhythmicity is starting to acquire solid bases and some of the molecular components of these clocks have now been identified. Interestingly, there are indications that some canonical circadian clock genes also contribute to the generation of ultradian and infradian rhythmicity. Pioneering work in this respect has been performed in marine organisms such as *Euridice pulchra* (Zhang et al., 2013) and *Platynereis dumerilii* (Zantke et al., 2013) for which tidal and circalunar clocks have been shown to control tide-related migration and gonadal maturation, respectively. In the marine midge *Clunio marinus*, moon light seems to play an important role in circalunar clock synchronization (Kaiser et al., 2016). All these clocks also exhibit some degree of independence, as they have been shown to function when the circadian clock is pharmacologically blocked (Zantke et al., 2013; Zhang et al., 2013). Further work is needed to define and functionally characterize the full set of components of such clocks, which represents one of the major current challenges in chronobiology.

In mammals, it has been observed that the phase differences between electrical and transcriptional/translational activity of neurons located in different SCN regions may reflect and thus code for the length of photoperiod (Inagaki et al., 2007; Yoshikawa et al., 2017; Honma, 2018). Such anatomical and functional organization may therefore provide organisms with relevant information to facilitate their adaptation to the environmental changes that characterize the course of seasons (circannual clock).

In humans, endogenous rhythmicity over any time scale is difficult to study, for reasons that are inherent to rhythms themselves and because of the masking/confounding effects of environmental cues to which we are sensitive, habits, social constraints etc. These can be removed only by complex and prolonged experiments [so-called constant routines (Duffy and Dijk, 2002)], which are generally performed in small numbers of young healthy individuals. Alternatively, rhythmicity can also be studied within the environment it is normally expressed in, in a sort of more ecological fashion, which yields somewhat less pure but still useful information. Further, while human circadian rhythmicity is fairly obvious, rhythms over different times scales are less apparent, and have not been the object of many studies. Of great interest, two sets of data have been recently published that provide examples of this non-circadian rhythmicity in humans. The first, which is the result of painstaking, patient and decades-long observations, has shown how women temporarily synchronize their menstrual cycles with the luminance and gravimetric cycles of the moon (Helfrich-Förster et al., 2021). The second one, which is the result of big data analysis, documents seasonality in human laboratory data collected for medical purposes, with a winter-spring peak in hormones related to reproduction, growth, metabolism, and stress adaptation (Tendler et al., 2021). It is not difficult to imagine how once the interest has focused on non-circadian human rhythmicity, both big data analyses of available datasets and the acquisition of new sets, for example by apps or other monitoring devices, will help producing information that is bound to be interesting and clinically relevant.

## Chronobiology and COVID-19

Infection from SARS-CoV-2 and the development of COVID-19 disease are very likely to affect circadian clock functioning. Moreover, rhythmicity over different time scales–most likely circadian and seasonal–may modulate the likelihood of acquisition and/or the course of infection and disease. Similarly, the intensive care arrhythmic environment may have unexpected effects on disease evolution (Haspel et al., 2021). The time of administration of approved treatments may impinge on the entity of their desired and side effects, in relation to both the nature of treatment itself, and to the patient's response to it (Haspel et al., 2021). These and other aspects of the complex and yet largely unexplored relationship between the pandemic and rhythmicity over different time scales have been considered by eminent colleagues in a collection of articles recently published in the Journal of Biological Rhythms (Sengupta et al., 2020, 2021; Borrmann et al., 2021; Cermakian and Harrington, 2021; Haspel et al., 2021; Kronfeld-Schor et al., 2021). As the pandemic continues to unfold, chronobiologists and scientists in related fields have become more sensitive to this relationship, and are examining available laboratory/clinical data retrospectively, and collecting them prospectively. Issues such as the appropriateness of time-stamping (clock time in addition to the full date) the acquisition of any human samples (may they be swabs, blood, urine etc.), the administration of treatment (Ruben et al., 2019) and the administration of vaccination for subsequent use in prognostic, large and long-term studies has once again come to the fore. Active, generous and powerful colleagues are lobbying on our behalf to this end.

Lockdowns put in place to different extents, in different countries and at different times of year have lead to some degree of stratification of society, with more fortunate groups enjoying some relief from social constraints and no major other changes to their lifestyle and productivity, and other groups being under considerable physical, emotional, organizational and economical stress, leading to a significant increase in mild and more severe psychiatric disorders (Holmes et al., 2020). There is an established relationship between psychiatric disease and rhythmicity, and the evidence for the benefits of chronotherapy (i.e., timed administration of light and/or melatonin) in this clinical field is considerably less anecdotal than generally perceived in other medical circles (Wirz-Justice and Benedetti, 2020).

At a time when all experience is needed to face the pandemic and its medium and long-term effects, translational chronobiology, chronopharmachology and chronotherapy, which also happens to be an inexpensive and substantially side-effect free form of treatment, may turn into powerful resources.

## Author Contributions

The author confirms being the sole contributor of this work and has approved it for publication.

## Conflict of Interest

The author declares that the research was conducted in the absence of any commercial or financial relationships that could be construed as a potential conflict of interest.

## References

[B1] Abruzzi K. C. Gobet C. Naef F. Rosbash M. (2021). Comment on “Circadian rhythms in the absence of the clock gene Bmal1”. Science 372:eabf0922. 10.1126/science.abf0922 33859000

[B2] Allen A. E. Martial F. P. Lucas R. J. (2019). Form vision from melanopsin in humans. Nat. Commun. 10:2274. 10.1038/s41467-019-10113-3 31118424 PMC6531428

[B3] Baño-Otálora B. Piggins H. D. (2017). Contributions of the lateral habenula to circadian timekeeping. Pharmacol. Biochem. Behav. 162, 46–54. 10.1016/j.pbb.2017.06.007 28624585

[B4] Bedont J. L. Iascone D. M. Sehgal A. (2020). The lineage before time: circadian and nonclassical clock influences on development. Annu. Rev. Cell Dev. Biol. 36, 469–509. 10.1146/annurev-cellbio-100818-125454 33021821 PMC10826104

[B5] Borrmann H. McKeating J. A. Zhuang X. (2021). The circadian clock and viral infections. J. Biol. Rhythms. 36, 9–22. 10.1177/0748730420967768 33161818 PMC7924106

[B6] Cederroth C. R. Albrecht U. Bass J. Brown S. A. Dyhrfjeld-Johnsen J. Gachon F.. (2019). Medicine in the fourth dimension. Cell Metab. 30, 238–250. 10.1016/j.cmet.2019.06.019 31390550 PMC6881776

[B7] Cermakian N. Harrington M. E. (2021). Chronobiology in response to COVID-19. J. Biol. Rhythms 36:3. 10.1177/0748730421993352 33543668

[B8] Chen S. K. Badea T. C. Hattar S. (2011). Photoentrainment and pupillary light reflex are mediated by distinct populations of ipRGCs. Nature 476, 92–96. 10.1038/nature10206 21765429 PMC3150726

[B9] Cuddapah V. A. Zhang S. L. Sehgal A. (2019). Regulation of the Blood–Brain Barrier by Circadian Rhythms and Sleep. Trends Neurosci. 42, 500–510. 10.1016/j.tins.2019.05.001 31253251 PMC6602072

[B10] Duffy J. F. Dijk D. J. (2002). Getting through to circadian oscillators: why use constant routines? J. Biol. Rhythms. 17, 4–13. 10.1177/074873002129002294 11837947

[B11] Edgar R. S. Green E. W. Zhao Y. Van Ooijen G. Olmedo M. Qin X.. (2012). Peroxiredoxins are conserved markers of circadian rhythms. Nature 485, 459–464. 10.1038/nature11088 22622569 PMC3398137

[B12] Fernandez D. C. Chang Y. T. Hattar S. Chen S. K. (2016). Architecture of retinal projections to the central circadian pacemaker. Proc. Natl. Acad. Sci. U.S.A. 113, 6047–6052. 10.1073/pnas.1523629113 27162356 PMC4889372

[B13] Haspel J. Kim M. Zee P. Schwarzmeier T. Montagnese S. Panda S.. (2021). A timely call to arms: COVID-19, the circadian clock, and critical care. J. Biol. Rhythms 36:074873042199258. 10.1177/0748730421992587 33573430 PMC7882674

[B14] Hastings M. H. Maywood E. S. Brancaccio M. (2019). The mammalian circadian timing system and the suprachiasmatic nucleus as its pacemaker. Biology 8:13. 10.3390/biology8010013 30862123 PMC6466121

[B15] Hattar S. Liao H. W. Takao M. Berson D. M. Yau K. W. (2002). Melanopsin-containing retinal ganglion cells: architecture, projections, and intrinsic photosensitivity. Science 295, 1065–1070. 10.1126/science.1069609 11834834 PMC2885915

[B16] Helfrich-Förster C. Monecke S. Spiousas I. Hovestadt T. Mitesser O. Wehr T. A. (2021). Women temporarily synchronize their menstrual cycles with the luminance and gravimetric cycles of the Moon. Sci. Adv. 7:eabe1358. 10.1126/sciadv.abe1358 33571128 PMC7840133

[B17] Holmes E. A. O'Connor R. C. Hugh Perry V. Tracey I. Wessely S. Arseneault L.. (2020). Multidisciplinary research priorities for the COVID-19 pandemic: a call for action for mental health science. Lancet Psychiatry 7, 547–560. 10.1016/S2215-0366(20)30168-1 32304649 PMC7159850

[B18] Honma S. (2018). The mammalian circadian system: a hierarchical multi-oscillator structure for generating circadian rhythm. J. Physiol. Sci. 68, 207–219. 10.1007/s12576-018-0597-5 29460036 PMC10717972

[B19] Inagaki N. Honma S. Ono D. Tanahashi Y. Honma K. (2007). Separate oscillating cell groups in mouse suprachiasmatic nucleus couple photoperiodically to the onset and end of daily activity. Proc. Natl. Acad. Sci. U.S.A. 104, 7664–7669. 10.1073/pnas.0607713104 17463091 PMC1857228

[B20] Kaiser T. S. Poehn B. Szkiba D. Preussner M. Sedlazeck F. J. Zrim A.. (2016). The genomic basis of circadian and circalunar timing adaptations in a midge. Nature. 540, 69–73. 10.1038/nature20151 27871090 PMC5133387

[B21] Ko G. Y. P. (2020). Circadian regulation in the retina: from molecules to network. Eur. J. Neurosci. 51, 194–216. 10.1111/ejn.14185 30270466 PMC6441387

[B22] Kronfeld-Schor N. Stevenson T. J. Nickbakhsh S. Schernhammer E. S. Dopico X. C. Dayan T.. (2021). Drivers of infectious disease seasonality: potential implications for COVID-19. J. Biol. Rhythms 36, 35–54. 10.1177/0748730420987322 33491541 PMC7924107

[B23] Mizrak D. Ruben M. Myers G. N. Rhrissorrakrai K. Gunsalus K. C. Blau J. (2012). Electrical activity can impose time of day on the circadian transcriptome of pacemaker neurons. Curr. Biol. 22, 1871–1880. 10.1016/j.cub.2012.07.070 22940468 PMC3562355

[B24] Montaigne D. Marechal X. Modine T. Coisne A. Mouton S. Fayad G.. (2018). Daytime variation of perioperative myocardial injury in cardiac surgery and its prevention by Rev-Erbα antagonism: a single-centre propensity-matched cohort study and a randomised study. Lancet 391, 59–69. 10.1016/S0140-6736(17)32132-3 29107324

[B25] Nakajima M. Imai K. Ito H. Nishiwaki T. Murayama Y. Iwasaki H.. (2005). Reconstitution of circadian oscillation of cyanobacterial KaiC phosphorylation *in vitro*. Science 308, 414–415. 10.1126/science.1108451 15831759

[B26] Ness-Cohn E. Allada R. Braun R. (2021). Comment on “Circadian rhythms in the absence of the clock gene Bmal1”. Science 372:eabe9230. 10.1126/science.abe,9230 33859007 PMC9172996

[B27] O'Neill J. S. Reddy A. B. (2011). Circadian clocks in human red blood cells. Nature 469, 498–504. 10.1038/nature,09702 21270888 PMC3040566

[B28] O'Neill J. S. Van Ooijen G. Dixon L. E. Troein C. Corellou F. Bouget F. Y.. (2011). Circadian rhythms persist without transcription in a eukaryote. Nature 469, 554–558. 10.1038/nature09654 21270895 PMC3040569

[B29] Panda S. Provencio I. Tu D. C. Pires S. S. Rollag M. D. Castrucci A. M.. (2003). Melanopsin is required for non-image-forming photic responses in blind mice. Science 301, 525–527. 10.1126/science.1086179 12829787

[B30] Provencio I. Jiang G. De Grip W. J. Pär Hayes W. Rollag M. D. (1998). Melanopsin: an opsin in melanophores, brain, and eye. Proc. Natl. Acad. Sci. U.S.A. 95, 340–345. 10.1073/pnas.95.1.340 9419377 PMC18217

[B31] Provencio I. Rollag M. D. Castrucci A. M. (2002). Photoreceptive net in the mammalian retina. Nature 415:493. 10.1038/415493a 11823848

[B32] Ray S. Valekunja U. K. Stangherlin A. Howell S. A. Snijders A. P. Damodaran G.. (2020). Circadian rhythms in the absence of the clock gene Bmal1. Science 367, 800–806. 10.1126/science.aaw7365 32054765

[B33] Roenneberg T. Wirz-Justice A. Skene D. J. Ancoli-Israel S. Wright K. P. Dijk D. J.. (2019). Why should we abolish daylight saving time? J. Biol. Rhythms 34, 227–230. 10.1177/0748730419854197 31170882 PMC7205184

[B34] Ruben M. D. Francey L. J. Guo Y. Wu G. Cooper E. B. Shah A. S.. (2019). A large-scale study reveals 24-h operational rhythms in hospital treatment. Proc. Natl. Acad. Sci. U.S.A. 116, 20953–20958. 10.1073/pnas.1909557116 31575744 PMC6800314

[B35] Ruby N. F. Brennan T. J. Xie X. Cao V. Franken P. Heller H. C.. (2002). Role of melanopsin in circadian responses to light. Science 298, 2211–2213. 10.1126/science.1076701 12481140

[B36] Sengupta S. Brooks T. G. Grant G. R. FitzGerald G. A. (2020). Accounting for time: circadian rhythms in the time of COVID-19. J. Biol. Rhythms 36, 4–8. 10.1177/0748730420953335 32875944 PMC8685580

[B37] Sengupta S. Ince L. Sartor F. Borrmann H. Zhuang X. Naik A.. (2021). Clocks, viruses, and immunity: lessons for the COVID-19 pandemic. J. Biol. Rhythms 36, 23–34. 10.1177/0748730420987669 33480287 PMC7970201

[B38] Tendler A. Bar A. Mendelsohn-Cohen N. Karin O. Korem Kohanim Y. Maimon L.. (2021). Hormone seasonality in medical records suggests circannual endocrine circuits. Proc. Natl. Acad. Sci. U.S.A. 118:e2003926118. 10.1073/pnas.2003926118 33531344 PMC7896322

[B39] Wirz-Justice A. Benedetti F. (2020). Perspectives in affective disorders: clocks and sleep. Eur. J. Neurosci. 51, 346–365. 10.1111/ejn.14362 30702783

[B40] Yoshikawa T. Inagaki N. F. Takagi S. Kuroda S. Yamasaki M. Watanabe M.. (2017). Localization of photoperiod responsive circadian oscillators in the mouse suprachiasmatic nucleus. Sci. Rep. 7:8210. 10.1038/s41598-017-08186-5 28811515 PMC5557852

[B41] Zantke J. Ishikawa-Fujiwara T. Arboleda E. Lohs C. Schipany K. Hallay N.. (2013). Circadian and circalunar clock interactions in a marine annelid. Cell Rep. 5, 99–113. 10.1016/j.celrep.2013.08.031 24075994 PMC3913041

[B42] Zhang L. Hastings M. H. Green E. W. Tauber E. Sladek M. Webster S. G.. (2013). Dissociation of circadian and circatidal timekeeping in the marine crustacean Eurydice pulchra. Curr. Biol. 23, 1863–1873. 10.1016/j.cub.2013.08.038 24076244 PMC3793863

